# Supplementary material for: Autophagy in Rat Müller Glial Cells Is Modulated by the Sirtuin 4/AMPK/mTOR Pathway and Induces Apoptosis under Oxidative Stress
Source: Cells. 2022 Aug 25;11(17):2645. doi: 10.3390/cells11172645 (PMC9454555; doi:10.3390/cells11172645)
Supplement: Supplementary file 1 [file cells-11-02645-s001.zip › cells-1799942-supplementary.pdf]

Supplementary Information for

Autophagy in rat Müller glia cell is modulated by Sirtuin4/AMPK/mTOR pathway and induce apoptosis under oxidative stress

Meng-Qi Qin†, Zhi Xie†, Ting Cao, Zhi-Ruo Wang, Xiao-Yu Zhang, Fei-Fei Wang , Wei Wei, Ming Jin, Jing-Yuan Ma, Zeng Lin, Ya-Nan Wang, Shao-Nan Pei and Xu Zhang*

Correspondence: Xu Zhang

E-mail: xuzhang19@163.com

This PDF file includes:

FIGURE S1-S15

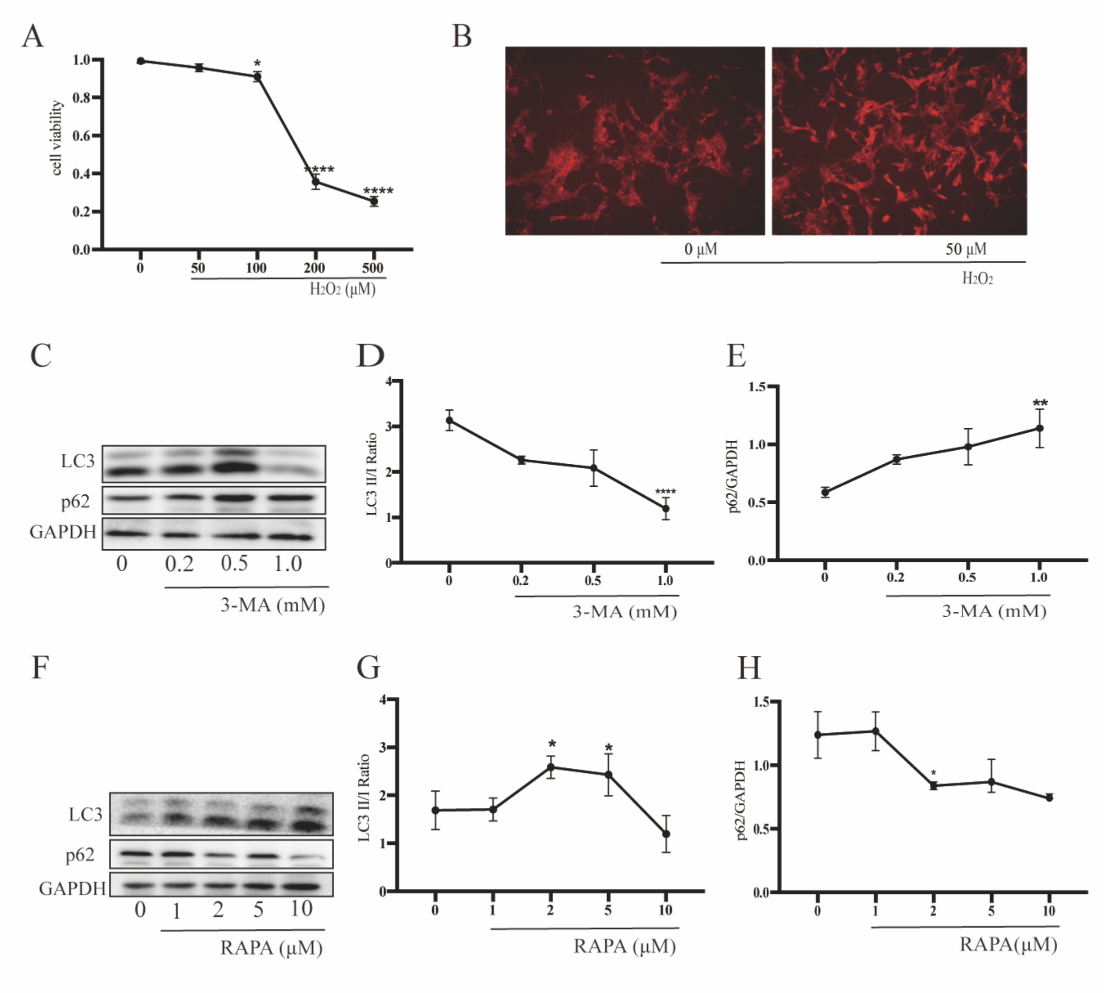

FIGURE S1: The subtoxic level of H_2O_2 and optimal concentration of 3-MA and rapamycin. (Sup.1 A) CCK8 were performed to detect cell viability under different H_2O_2 doses. The MGC were exposed to the H_2O_2 at 0, 50, 100, 150, 200μM respectively for 24h, and found that exposure of MGC to 50μM did not lead to loss of cell viability. (Sup.1 B) the ROS generation in r-MC under normal condition or 50μM H_2O_2 was detected by DHE kit via immunofluorescence. (Sup.1 C-E)The quantitative analysis of LC3 and SQSTM1/p62 were performed to detect optimal concentration of 3-MA. Data are shown as mean ± SEM. (n=3 per group, ***P*<0.01, *****P*<0.0001). The autophagic response which is determined by the change in expression of LC3 and SQSTM1/p62. Western blot analysis showed that 3-MA can significantly repress autophagy function at concentration of 1mM. (Sup.1 F-H)The quantitative analysis of LC3 and SQSTM1/p62 were performed to detect optimal concentration of rapamycin. Data are shown as mean ± SEM. (n=3 per group, *P<0.05) The autophagic response which is determined by the change in expression of LC3 and SQSTM1/p62. Western blot analysis showed that rapamycin can significantly enhance autophagy function at concentration of 2μM. (p62, SQSTM1/p62)

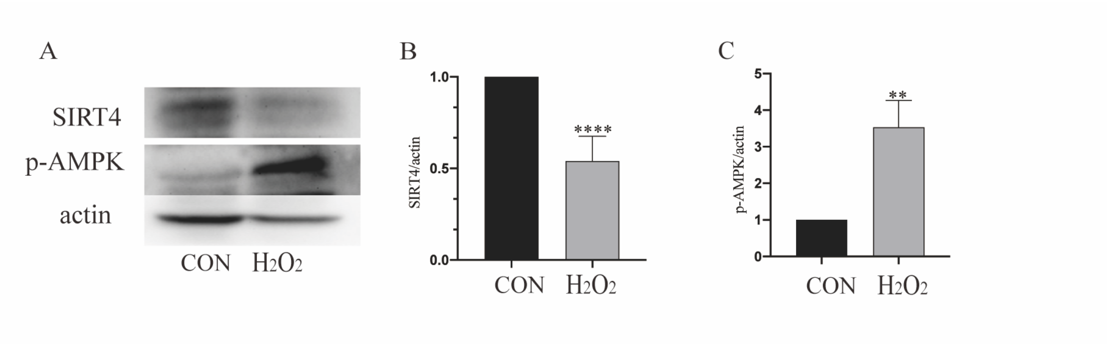

FIGURE S2: 50μM H_2O_2 can inhibit SIRT4 and activate p-AMPK expression in Müller glial cells. (A)The expressional level of SIRT4 and p-AMPK with or without 50μM H_2O_2 treatment in Müller glial cells. (B) SIRT4 is downregulated under 50μM H_2O_2. Data are shown as mean ± SEM. (n=3 per group, ****P<0.0001). (C) p-AMPK is activated under 50μM H_2O_2. Data are shown as mean ± SEM. (n=3 per group, **P<0.01).

FIGURE S3

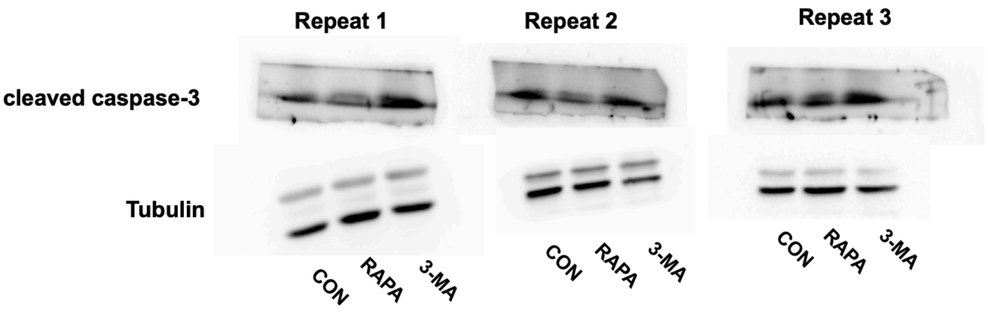

Original western images used for preparing FIGURE 1B

FIGURE S4

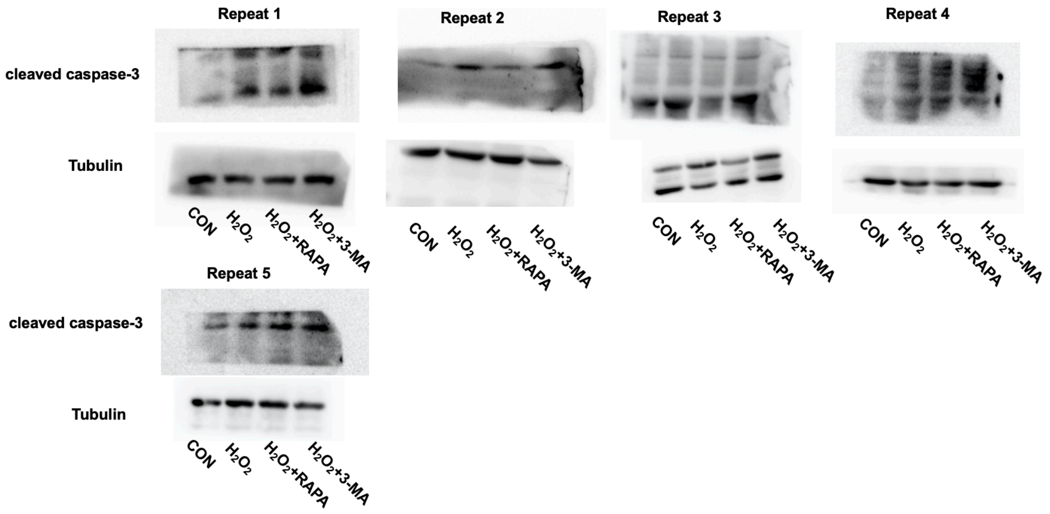

Original western images used for preparing FIGURE 1C

FIGURE S5

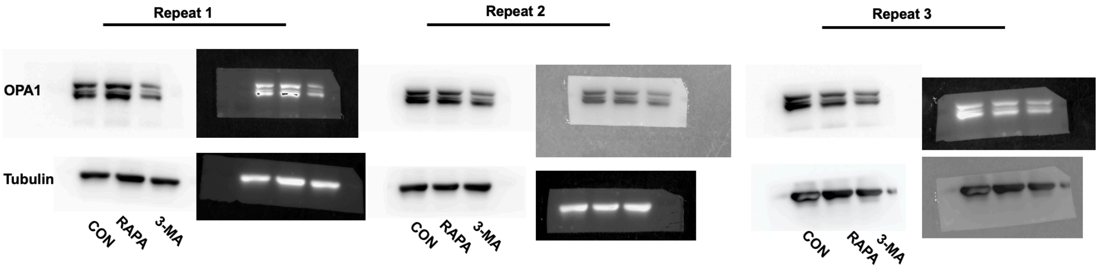

Original western images used for preparing FIGURE 2E

FIGURE S6

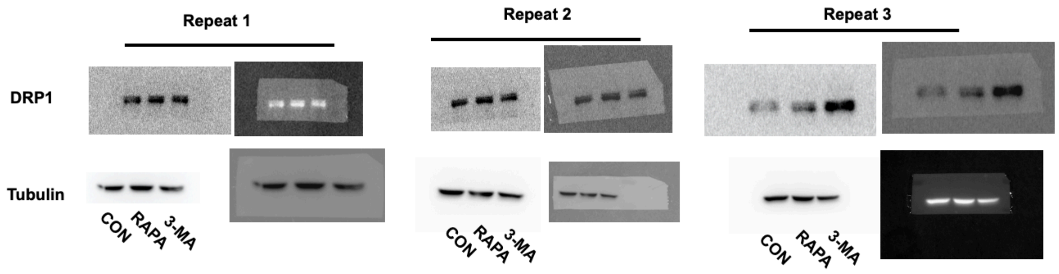

Original western images used for preparing FIGURE 2F

FIGURE S7

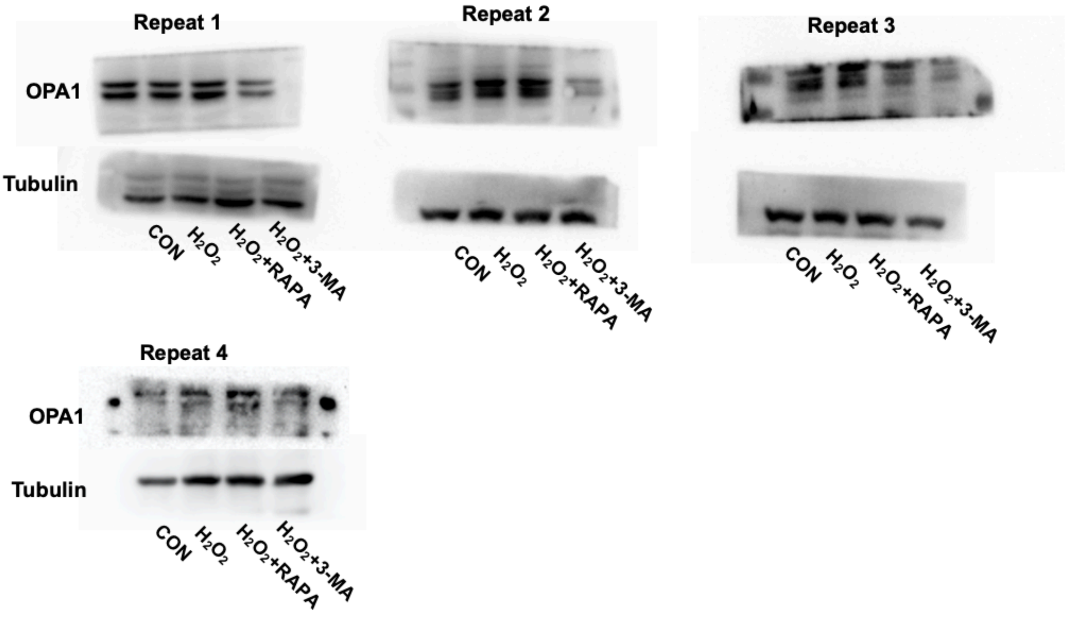

Original western images used for preparing FIGURE 2G

FIGURE S8

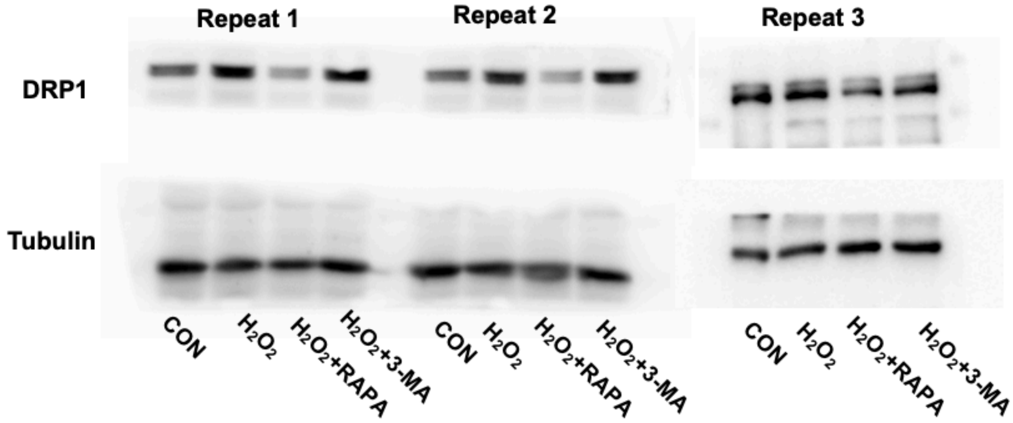

Original western images used for preparing FIGURE 2H

FIGURE S9

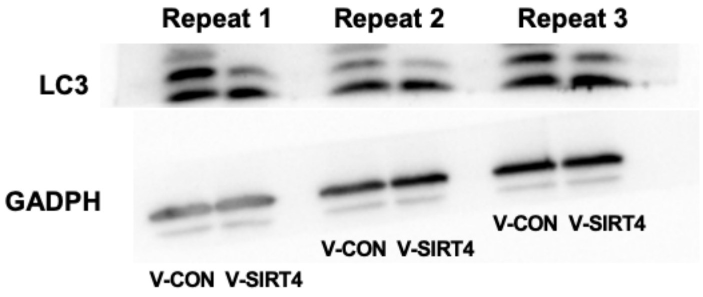

Original western images used for preparing Figure 6 H

FIGURE S10

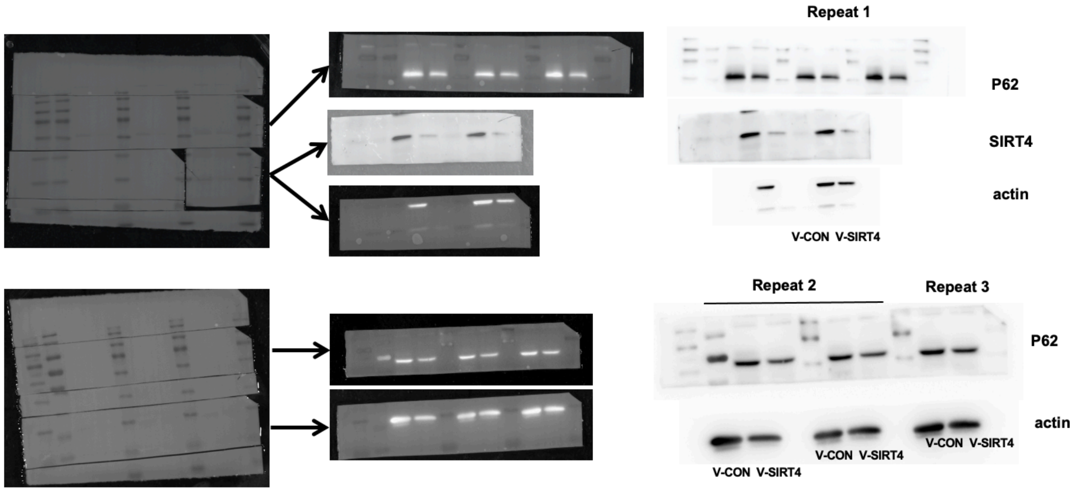

Original western images used for preparing FIGURE 6 I

FIGURE S11

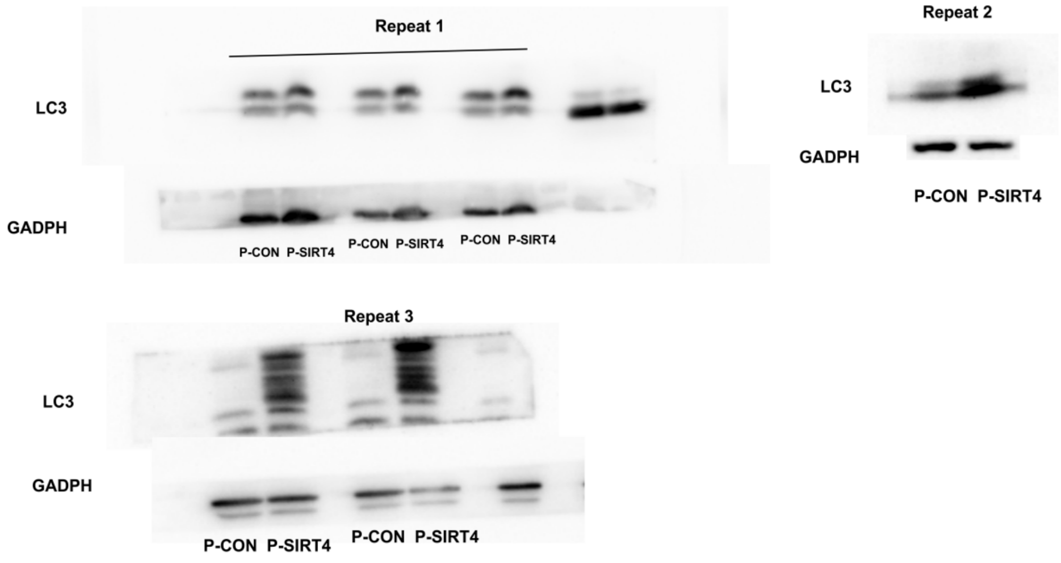

Original western images used for preparing Figure 6E

FIGURE S12

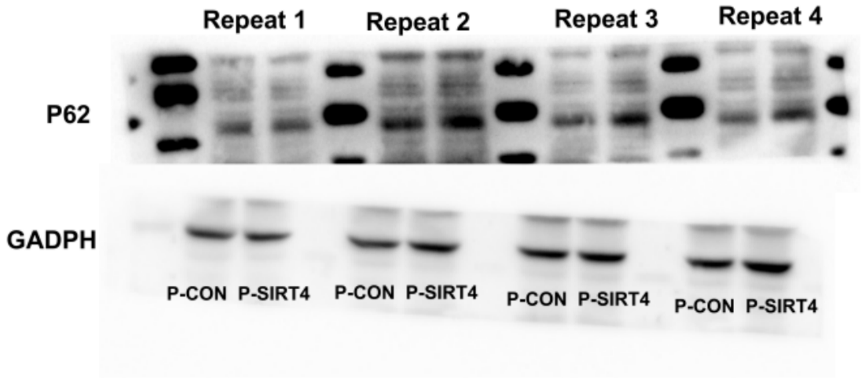

Original western images used for preparing Figure 6F

FIGURE S13

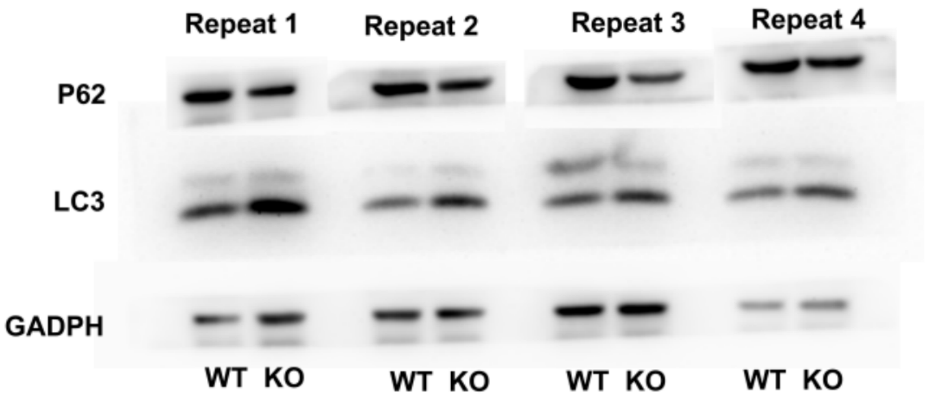

Original western images used for preparing FIGURE 6 B - C

FIGURE S14

Repeat 1

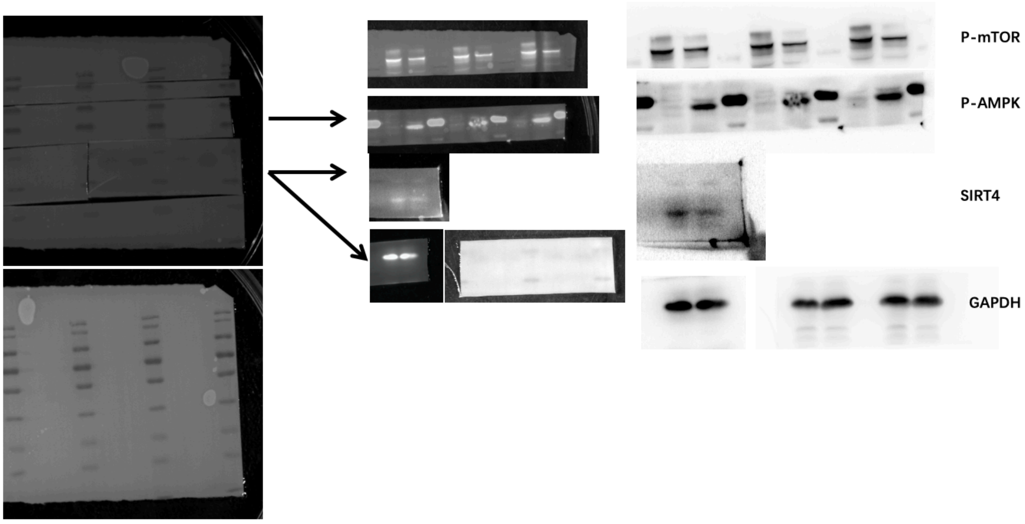

Repeat 2

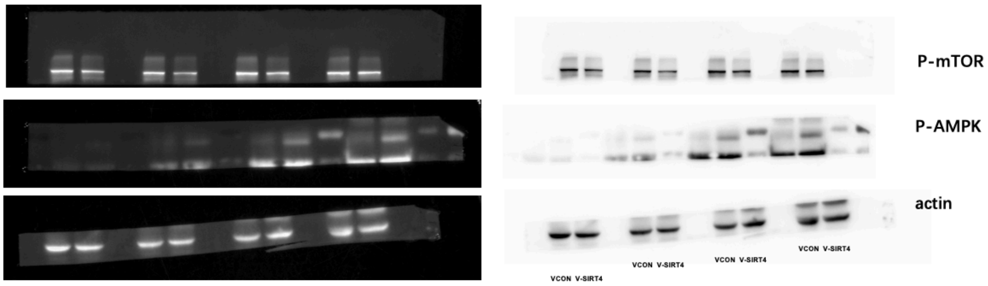

Repeat 3 **Repeat 4**

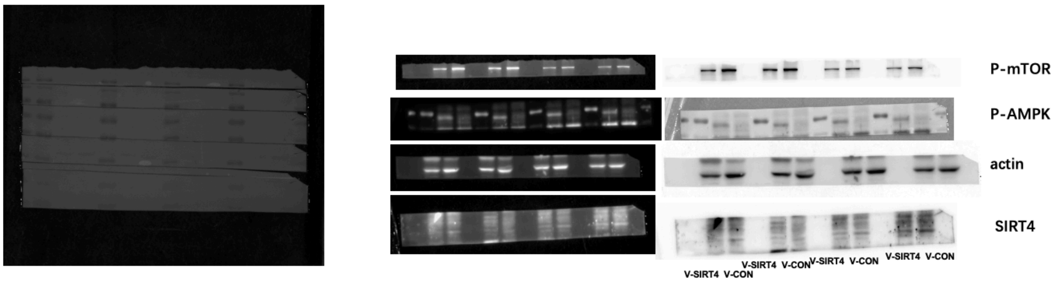

Original western images used for preparing FIGURE 7A-B

FIGURE S15

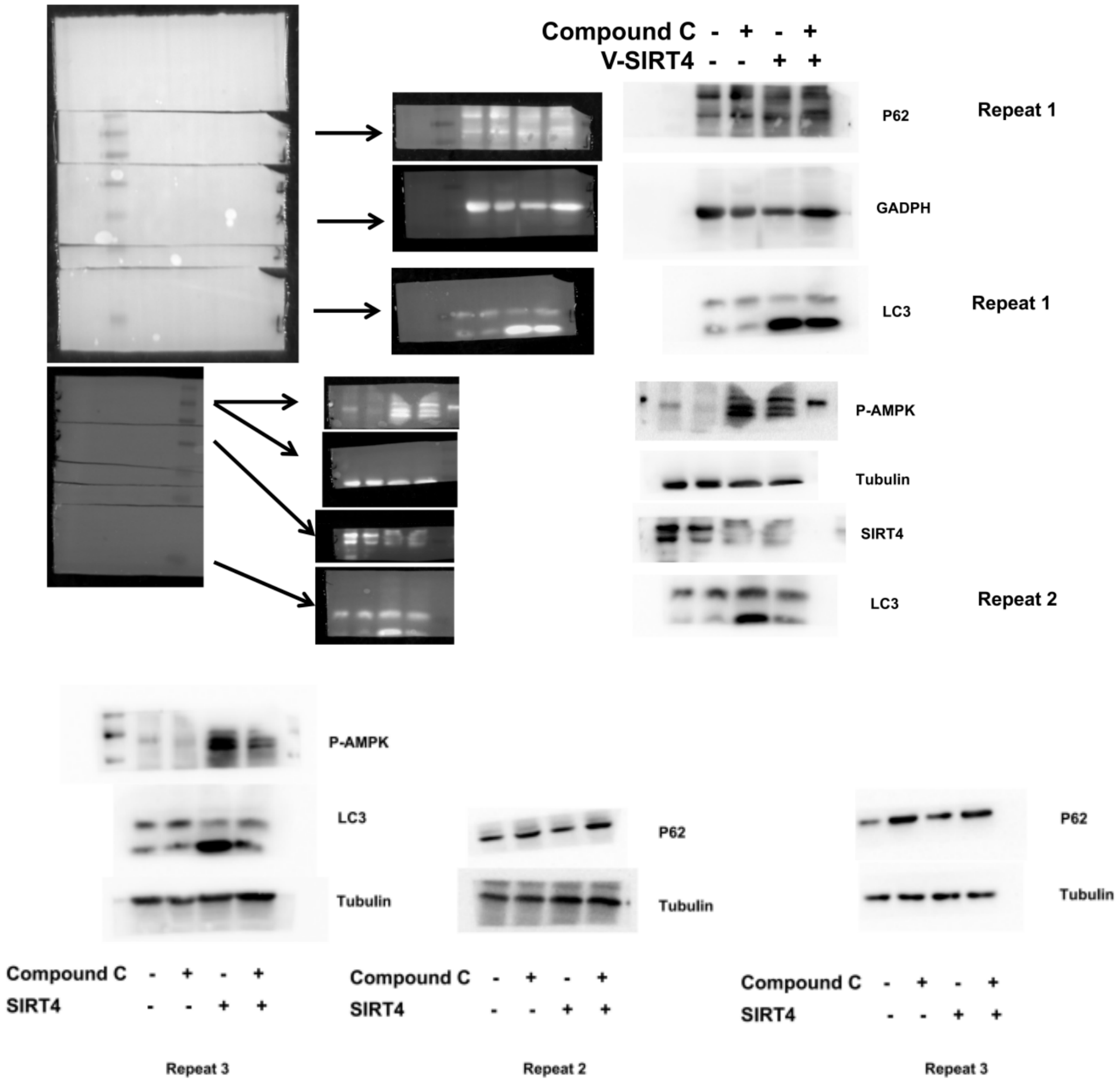

Original western images used for preparing Figure 7 D -F
